# Characterization of ten white matter tracts in a representative sample of Cuban population

**DOI:** 10.1186/s12880-016-0163-7

**Published:** 2016-10-26

**Authors:** D. Góngora, M. Domínguez, M. A. Bobes

**Affiliations:** 1 Key Laboratory for NeuroInformation of Ministry of Education, Center for Information in Medicine, University of Electronic Science and Technology of China, 2006, Xiyuan Ave, West Hi-Tech Zone, Chengdu, 61000 China; 2 Cuban Neuroscience Center, 190th Ave between 25th and 27th Ave, Havana, 11300 Cuba; 3 IDIBELL Bellvitge Biomedical Research Institute, Barcelona, Spain

**Keywords:** Diffusion tensor imaging, Volume, Fractional anisotropy, Mean diffusivity, White matter tracts

## Abstract

**Background:**

The diffusion tensor imaging technique (DTI) combined with tractography methods, has achieved the tridimensional reconstruction of white matter tracts in the brain. It allows their characterization in vivo in a non-invasive way. However, one of the largest sources of variability originates from the location of regions of interest, is therefore necessary schemes which make it possible to establish a protocol to be insensitive to variations in drawing thereof. The purpose of this paper is to stablish a reliable protocol to reconstruct ten prominent tracts of white matter and characterize them according to volume, fractional anisotropy and mean diffusivity. Also we explored the relationship among these factors with gender and hemispheric symmetry.

**Methods:**

This study aims to characterize ten prominent tracts of white matter in a representative sample of Cuban population using this technique, including 84 healthy subjects. Diffusion tensors and subsequently fractional anisotropy and mean diffusivity maps were calculated from each subject’s DTI scans. The trajectory of ten brain tracts was estimated by using deterministic tractography methods of fiber tracking. In such tracts, the volume, the FA and MD were calculated, creating a reference for their study in the Cuban population. The interactions between these variables with age, cerebral hemispheres and gender factors were explored using Repeated Measure Analysis of Variance.

**Results:**

The volume values showed that a most part of tracts have bigger volume in left hemisphere. Also, the data showed bigger values of MD for males than females in all the tracts, an inverse behavior than FA values.

**Conclusions:**

This work showed that is possible reconstruct white matter tracts using a unique region of interest scheme defined from standard to native space. Also, this study indicates differing developmental trajectories in white matter for males and females and the importance of taking gender into account in developmental DTI studies and in underlie gender-related cognitive differences.

**Electronic supplementary material:**

The online version of this article (doi:10.1186/s12880-016-0163-7) contains supplementary material, which is available to authorized users.

## Background

The ability to identify and characterize the nerve fiber tracts that connect nodes (functional areas) is crucial to advance the understanding of brain function in both normal and pathological conditions. Nuclear Magnetic Resonance conventional techniques are able to distinguish specific white matter tracts only in small and restricted areas of the brain [1, 2]. However, using Diffusion Tensor Images (DTI) and processing techniques developed for the layout of the fibers, it has been possible to delineate and reconstruct three-dimensional fiber tracts of white matter, with a good agreement with anatomical data [3, 4]. This procedure, known as tractography, has opened a new window on the important topic of brain connectivity [5].

Based on DTI and applying a deterministic method dubbed Fiber Assignment by Continuous Tracking (FACT), the tractography allows the approximated reconstruction of white matter fibers, advancing from voxel by voxel, according to an estimate of the local orientation of nerve fibers [4]. Here, the aforementioned estimation of the fiber path stops when it reaches the outer volume limits, a region where fractional anisotropy or some index of inter-voxel coherence is less than certain threshold values for which the uncertainty is considered high by taking a direction to follow, or up to a pre-selected region of interest [6].

The FACT method allows a good characterization of white matter tracts. In healthy subjects, this characterization is required to permit patterns analysis of brain connectivity, and to make comparisons with pathological conditions. The main white matter tracts have been successfully reconstructed in healthy subjects. However, it was used small samples (less than 30 subjects), mostly Caucasian populations without emphasis in variables that characterize the peculiarities of each tract [7–9].

In tractography, one of the largest sources of variability originates from the location of regions of interest (ROIs). ROIs are defined a priori as anatomical regions from which identify specific tracts [10]. It is therefore necessary ROIs schemes which make it possible to establish a protocol to be insensitive to variations in drawing thereof. This problem has been addressed in previous studies that have defined a set of tract-specific ROIs allowing the reproducible reconstruction of white matter tracts [7–9].

Practical applications of tractography have unquestionable value, for example in case of neurosurgery, where it provides guidance in preoperative planning [11, 12]. In this regard, to establish and validate reconstruction procedures of trajectories of white matter tracts in humans, reproducible between subjects, is crucial to allow an understandable use of this technique.

Given this background and the fact that there are variations in brain anatomy in terms of factors such as hemispheric symmetry, gender and studied population [13], the purpose of this paper is to reconstruct ten prominent tracts of white matter and characterize them according to volume, Fractional Anisotropy (FA) and Mean Diffusivity (MD). Also we explored the relationship among these factors with gender and hemispheric symmetry.

## Methods

### Sample

The sample included 84 healthy subjects who are part of the Cuban Project of Human Brain Mapping. This sample was made up of randomly selected subjects of the population of the municipality of La Lisa, Havana. This population is considered representative in terms of ethnic and gender distribution of the Cuban population. Participants were included in the study after reading, accepting and sign an informed consent, in accordance with the ethical standards of the Declaration of Helsinki [14], and the experimental protocols were approved by the Ethics Committee of Cuban Neuroscience Center. A statistical description of the sample is presented in Table 1.

**Table 1:** Statistical description of the sample

	Subjects (n)	School level^a^	Age^a^	Intelligence quotient^a^
Women	44	12.44 ± 2.46	38.75 ± 9.95	91.14 ± 11.87
Men	40	11.78 ± 2.44	31.00 ± 8.97	90.72 ± 11.44
Total	84	12.12 ± 2.46	35.06 ± 10.21	90.94 ± 11.60

^a^Mean values ± standard deviation

The total sample consisted of right handed subjects with an intelligence quotient (IQ) within the range reported as normal. IQ was obtained for each subject using the Spanish language version of the Wechsler Adult Intelligence III Scale [15].

Each subject underwent an interview and medical examination with Neurology and Psychiatry specialists, in order to rule out any pathology of the nervous system to invalidate their participation in the study. Neurological examination was performed following the procedure described in guidelines published by the Department of Health and Human Services U.S. in 2003. Mini-International Psychiatric Interview was used for psychiatric evaluation [16].

### Acquisition of images

Using a scanner Siemens Symphony 1.5 T (Erlangen, Germany) was acquired for each subject a T1 anatomical image of high resolution 3D, and a standard scheme of diffusion gradients. The T1 anatomical image was recorded with the following characteristics: 160 contiguous sagittal slices 1 mm thick, field of view (FOV) = 256 × 256 mm^2^, corresponding to a resolution in sagittal plane of 1 × 1 mm^2^, echo time (ET) = 3.93 ms, repetition time (RT) = 3000 ms. Using a single echo planar imaging (EPI) sequence, twelve diffusion-weighted images were obtained (b = 1200 s/mm^2^) and a reference T2 weighted image (b0 image) with no diffusion weighting (b = 0 s/mm^2^). The acquisition parameters were: FOV = 256 × 256 mm^2^, acquired matrix = 128 × 128, corresponding to a resolution in the axial plane of 2 × 2 mm^2^, ET/RT = 160/7000 ms. The slice number was adapted to cover the whole brain with a slice thickness of 3 mm. The acquisition scheme was repeated 5 times to average the corresponding images and thus improving the signal/noise ratio.

In order to correct the distortions caused by magnetic field inhomogeneities in the series of diffusion-weighted images, phase and magnitude maps were obtained. The parameters used were: voxel size of 3.5 mm; ET_1_ = 7.71 ms, ET_2_ = 12.47 ms and RT = 672 ms.

### Processing diffusion weighted images

On b0 images was detected the presence of Gibbs artifacts around the ventricles. To correct these artifacts a Hanning filter was applied to these images. Then, Eddy currents correction was made by linear recording of weighted images to b0. Using the images of magnitude and phase, and Unwarping package of SPM5 program (http://www.fil.ion.ucl.ac.uk/spm/) the effects of main magnetic field inhomogeneities were corrected [17].

### Estimation of the diffusion tensor and fiber tracking

The toolbox DTI & Fiber Tools v.3.0 [18] was utilized to estimate at each voxel the six elements of the diffusion tensor as formulated by Basser et al. [19]. After tensor diagonalization, three eigenvalues and eigenvectors were obtained and calculated FA and MD maps.

Three-dimensional reconstruction of the tracts was performed using FACT, a deterministic tractography method [4]. The parameters used in tracts reconstruction were for the beginning of traced FA threshold = 0.15 and Trace = 0.0016, and as a stop criteria FA = 0.10, Trace = 0.002 and maximum bending angle of 53.1 °.

The fiber tracking was performed in all brain voxels, and fibers that penetrated the previously defined ROIs were assigned to specific tracts associated with them. ROIs were defined for the following tracts: anterior thalamic radiation (ATR), cingulate gyrus associated cingulum (CGC), hippocampal gyrus associated cingulum (CGH), cortico-spinal tract (CST), inferior fronto-occipital fasciculus (IFOF), inferior longitudinal fasciculus (ILF), superior longitudinal fasciculus (SLF), uncinate fasciculus (UNC), forceps major (Fmj) and forceps minor (Fmn). The resulting path of these tracts were visually inspected and corrected in cases where necessary, by the exclusion of fibers that do not belongs anatomically to tracts.

### Definition of ROIs in standard space and space transformation procedure to each subject anatomical space

Definition of ROIs for studied tracts was made by replicating a set of predefined ROI by Mori et al. [8] that was employed successfully in subsequent work [7, 9, 20, 21]. These ROIs were drawn using the program MRIcron (http://www.mccauslandcenter.sc.edu/crnl/mricron/) on a reference anatomical image with spatial resolution of 1 × 1 × 1 mm^3^ in stereotactic space of the Montreal Neurological Institute (MNI) [22]. The ROIs were then transformed to each individual brain space automatically, using a programmed routine in MatLab v.7.7 (MathWorks, Inc.).

### Parameter estimation

Voxels that conformed each one of the estimated tracts were extracted. The volume of each tract was estimated by multiplication of the total number of voxels of each tract by a voxel volume (0.012 cm^3^). The FA and MD was obtained as an estimate of the average in each tract, which resulted from the superposition of the specific coordinates for each tract on the corresponding maps of FA and MD.

### Statistical analysis

The tracts were explored according to volume, FA and MD values using the Statistica software v.10.0 (StatSoft, Inc.) and was considered a level of significance of α = 0.05 in all cases. The differences between hemispheres and gender were assessed using the General Lineal Model (GLM) for Repeated Measure Analysis of Variance (rmANOVA), considering the factors Gender, Age, Tracts and Hemisphere; the last two factors were used as within effects, Gender as categorical predictor and Age as continuous predictor. A Greenhouse and Geisser [23] correction was applied. Planned comparisons were performed subsequently by specific contrasts. The inter-hemispheric tracts (Fmj and Fmn) were excluded from hemispheric asymmetries analysis.

## Results

### Reconstruction of the tracts of interest

The reconstruction of the tracts of interest was possible using the deterministic method FACT and ROIs obtained for each subject by the transformation proceeding of reconstruction of the trajectory proposed for ten tracts of interest in each of the 84 subjects enrolled in the study. These tracts were classified for description in four functional categories: brainstem fibers and projection, association fibers, tracts of the limbic system and commissural fibers (See Additional file 1).

### Characterization of reconstructed tracts

White matter tracts were characterized anatomically estimating the volume, FA and MD for each tract. The mean values in each tract are presented in Table 2. The average volume of studied tracts ranged from 5 to 38 cm^3^, in correspondence with the anatomical characteristics of each one. The FA values in the sample ranged from 0.34 to 0.58. The MD, meanwhile, it was distributed in a range of values from 0.54 × 10^−3^ to 0.7 × 10^−3^mm^2^s^−1^.

**Table 2:** Statistical description of Volume, FA and MD in the sample

		Volume (cm^3^)^a^	FA^a^	MD (×10^−3^mm^2^s^−1^)^a^
Anterior thalamic radiation (ATR)	Left	16.18 ± 6.04	0.44 ± 0.07	0.63 ± 0.14
	Right	11.93 ± 4.43	0.48 ± 0.08	0.59 ± 0.15
Cingulate gyrus associated cingulum (CGC)	Left	6.62 ± 3.00	0.42 ± 0.07	0.58 ± 0.16
	Right	5.42 ± 2.97	0.40 ± 0.05	0.60 ± 0.14
Hippocampal gyrus associated cingulum (CGH)	Left	5.69 ± 3.11	0.36 ± 0.09	0.68 ± 0.21
	Right	6.52 ± 4.30	0.35 ± 0.08	0.70 ± 0.19
Cortico-spinal tract (CST)	Left	5.93 ± 3.39	0.56 ± 0.08	0.64 ± 0.15
	Right	5.13 ± 3.07	0.58 ± 0.10	0.62 ± 0.18
Forceps major (Fmj)		22.98 ± 9.89	0.52 ± 0.06	0.61 ± 0.16
Forceps minor (Fmn)		38.27 ± 10.90	0.45 ± 0.07	0.62 ± 0.17
Inferior fronto-occipital fasciculus (IFOF)	Left	23.88 ± 8.59	0.48 ± 0.09	0.58 ± 0.16
	Right	26.96 ± 8.18	0.45 ± 0.07	0.60 ± 0.15
Inferior longitudinal fasciculus (ILF)	Left	12.59 ± 5.54	0.47 ± 0.09	0.59 ± 0.17
	Right	9.97 ± 4.57	0.46 ± 0.09	0.60 ± 0.15
Superior longitudinal fasciculus (SLF)	Left	16.87 ± 5.79	0.47 ± 0.09	0.54 ± 0.16
	Right	12.40 ± 6.22	0.46 ± 0.08	0.55 ± 0.15
Uncinate fasciculus (UNC)	Left	7.81 ± 3.37	0.37 ± 0.08	0.62 ± 0.17
	Right	7.11 ± 3.74	0.38 ± 0.07	0.63 ± 0.17

^a^Mean values ± standard deviation

### Gender differences and hemispheric asymmetries

In this paper we assessed if volume, FA and MD values had the same statistical behavior regarding hemisphere and gender using the GLM for rmANOVA, also the age was used as continuous predictor. However, no effect of age was found in the analysis. Subsequently, multiple comparisons were performed across specific contrasts of significant parameters in the model.

#### Volume

**Fig. 1:**
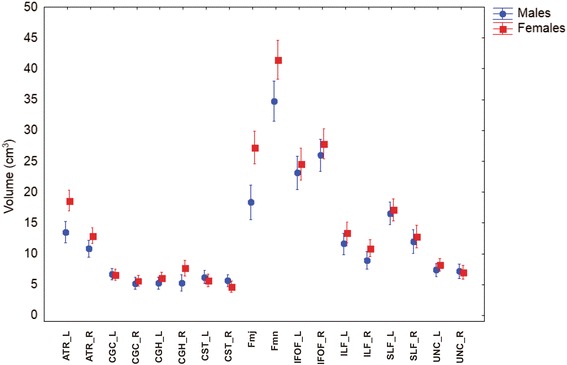
Volume of studied tracts by gender. *Vertical bars* denote 0.95 confidence intervals. (L: left hemisphere; R: right hemisphere)

The GLM Repeated Measure ANOVA using Tracts volume as repeated measures, Gender as categorical predictor and Age as continuous predictor showed that exist a main effect of Tracts volume (*F* = 26.40, df = 17, *p* < 0.001, ɛ = 0.425) and Gender (*F* = 11.98, df = 1, *p* = 0.001) (Fig. 1). The double interaction Tracts x Gender was significant (*F* = 5.929, df = 17, *p* < 0.001, ɛ = 0.425). The planned comparison analysis showed that the volume in female are significant larger than males for left ATR (F(1,81) = 22.366; *p* < 0.001), right ATR (F(1,81) = 7.958; *p* = 0.006), right CGH (F(1,81) = 5.609; *p* = 0.020), Fmj (F(1,81) = 21.654; *p* < 0.001) and Fmn (F(1,81) = 12.896; *p* < 0.001). The remaining tracts had larger volumes in females than males with no significant differences, with the exception of right CST and right UNC which presented larger volume for males (Fig. 1).

Excluding the commissural tracts, Fmj and Fmn, hemispheric asymmetries in volume were assessed by GLM Repeated Measure ANOVA using Tracts volume and Hemisphere as repeated measures, Gender as categorical predictor and Age as continuous predictor, showed that exist a main effect of Tracts (*F* = 19.69, df = 7, *p* < 0.001, ɛ = 0.618) and Hemisphere (*F* = 11.9, df = 1, *p* < 0.001). The double interaction Tracts x Hemisphere (*F* = 2.041, df = 7, *p* < 0.05) were significant. Planned comparison showed that the volume in the left hemisphere is larger than the right in both genders for the following tracts: ATR (F(1,81) = 52.461; *p* < 0.001), CGC (F(1,81) = 15.28; *p* < 0.001), CST (F(1,81) = 4.608; *p* = 0.035), ILF (F(1,81) = 18.968; *p* < 0,001) and SLF (F(1,81) = 34.558; *p* < 0.001).On the contrary, the volume in the right hemisphere was larger in CGH (F(1,81) = 5.624, *p* = 0.02) and the IFOF (F(1,81) = 12.377; *p* < 0,001). No significant differences between hemispheres in the volume of UNC were founded.

#### Fractional anisotropy

**Fig. 2:**
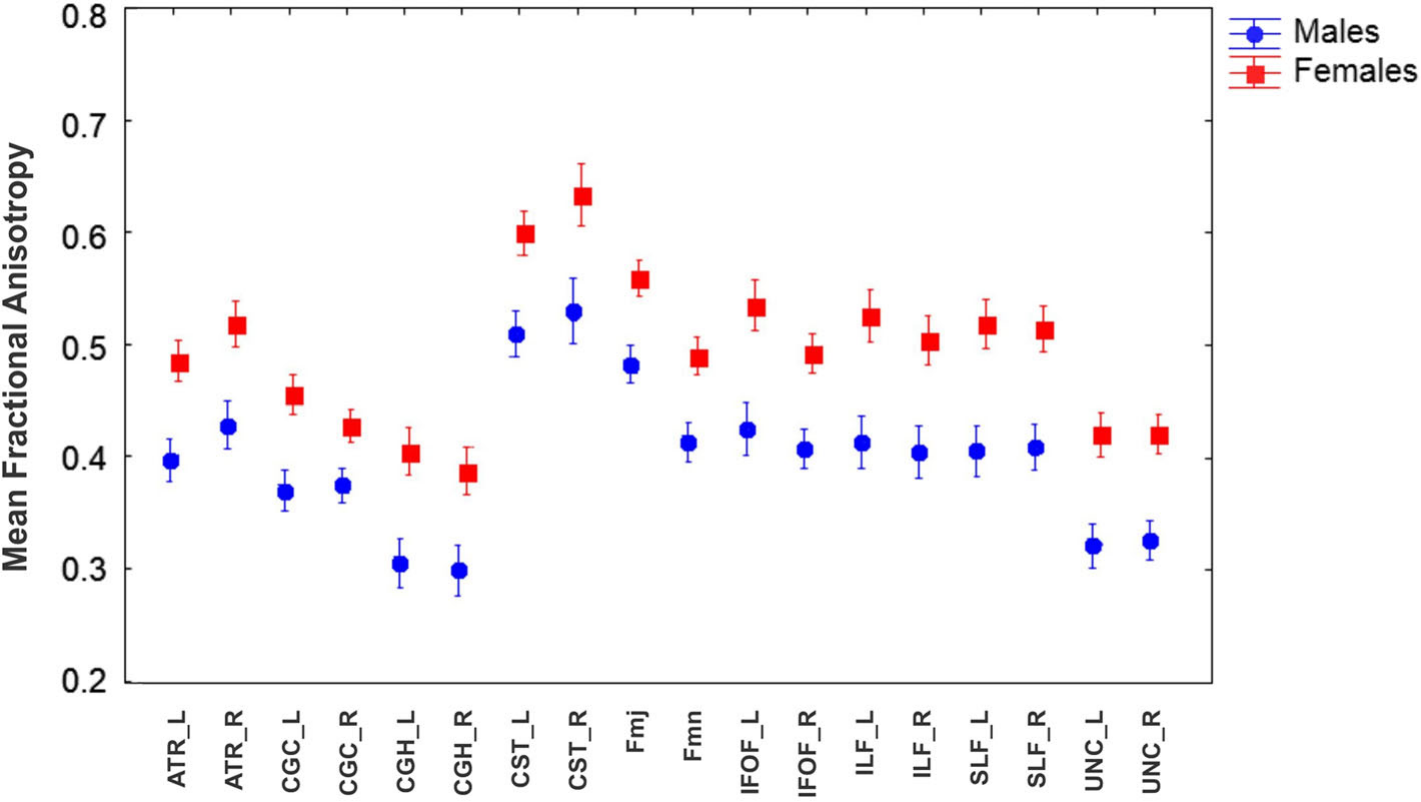
Mean fractional anisotropy of studied tracts by gender. *Vertical bars* denote 0.95 confidence intervals. (L: left hemisphere; R: right hemisphere)

The GLM Repeated Measure ANOVA using FA values (of each tract) as repeated measures, Gender as categorical predictor and Age as continuous predictor (Fig. 2) showed that exist a main effect of FA values (*F* = 10.489, df = 17, *p* < 0.001, ɛ = 0.562) and Gender (*F* = 47.31, df = 1, *p* < 0.001), with a significant interaction FA values x Gender (*F* = 4.15, df = 17, *p* < 0.001, ɛ = 0.569). The data showed bigger values of FA for females than males in all the tracts. By planned comparison analysis were found significant differences for left ATR (F(1,80) = 39.628; *p* < 0.001) and right ATR (F(1,80) = 33.143, *p* < 0.001), left CGC (F(1,80) = 38.909; *p* < 0.001) and right CGC (F(1,80) = 24.015; *p* < 0.001), left CGH (F(1,80) = 30.044; *p* < 0.001) and right CGH (F(1,80) = 31.322; *p* < 0.001), left CST (F(1,80) = 30.110; *p* < 0.001) and right CST (F(1,80) = 24.472; *p* < 0.001), Fmj (F(1,80) = 38.574; *p* < 0.001) and Fmn (F(1,80) = 40.929; *p* < 0.001), left IFOF (F(1,80) = 44.069; *p* < 0.001) and right IFOF (F(1,80) = 41.909; *p* < 0.001), left ILF (F(1,80) = 39.534; *p* < 0,001) and right ILF (F(1,80) = 34.545; *p* < 0.001), left SLF (F(1,80) = 45.333; *p* < 0.001) and right SLF (F(1,80) = 48.343; *p* < 0,001), left UNC (F(1,80) = 47.514; *p* < 0.001) and right UNC (F(1,80) = 46.738; *p* < 0.001) (Fig. 2).

Excluding the commissural tracts, Fmj and Fmn, hemispheric asymmetries in FA values were assessed by GLM Repeated Measure ANOVA using FA values (of each tract) and Hemisphere as repeated measures, Gender as categorical predictor and Age as continuous predictor showed that exist a main effect of FA (*F* = 28.629, df = 7, *p* < 0.001, ɛ = 0.769) and no effect for Hemisphere (*F* = 0.134, df = 1, *p* = 0.516, ɛ = 1); however, de double interaction FA values x Hemisphere was significant (*F* = 4.787, df = 7, *p* < 0.001, ɛ = 0.709). Planned comparison showed that CGC (F(1,80) = 8.237, *p* < 0.005), CGH (F(1,80) = 7.916; *p* < 0.005), IFOF (F(1,80) = 92.369; *p* < 0.001), ILF (F(1,80) = 7.492; *p* < 0.005) have a left asymmetry (FA in left hemisphere > FA in the right hemisphere). On the contrary ATR (F(1,80) = 115.629; *p* < 0.001) and CST (F(1,80) = 20.621; *p* < 0.001) showed a right asymmetry. No significant differences between hemispheres in the FA values of SLF and UNC were founded.

#### Mean diffusivity

**Fig. 3:**
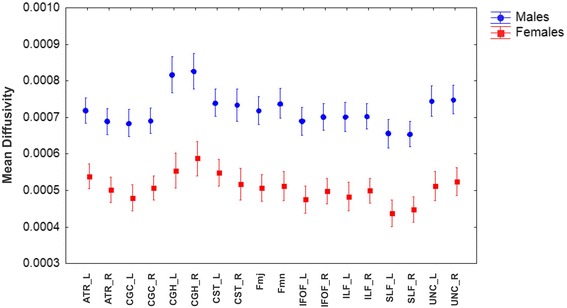
Mean diffusivity of studied tracts by gender. *Vertical bars* denote 0.95 confidence intervals. (L: left hemisphere; R: right hemisphere)

The GLM Repeated Measure ANOVA using MD values (of each tract) as repeated measures, Gender as categorical predictor and Age as continuous predictor (Fig. 3) showed that exist a main showed that exist a main effect of MD values (*F* = 10.069, df = 17, *p* < 0.001, ɛ = 0.382) and Gender (*F* = 55.40, df = 1, *p* < 0.001) with a significant interaction MD values x Gender (*F* = 9.000, df = 17, *p* < 0.001, ɛ = 0.382). The data showed bigger values of MD for males than females in all the tracts.

By planned comparison analysis were found significant differences for left ATR (F(1,80) = 48.650; p <0.001) and right ATR (F(1,80) = 50.375; *p* < 0.001), left CGC (F(1,80) = 54.193; *p* < 0.001) and right CGC (F(1,80) = 50.140; *p* < 0.001), left CGH (F(1,80) = 50.333; *p* < 0.001) and right CGH (F(1,80) = 43.136; *p* < 0.001), left CST (F(1,80) = 42.762; *p* < 0.001) and right CST (F(1,80) = 41.220; *p* < 0.001), Fmj (F(1,80) = 56.279; *p* < 0.001) and Fmn (F(1,80) = 57.800; *p* < 0.001), left IFOF (F(1,80) = 55.654; *p* < 0.001) and right IFOF (F(1,80) = 56.077; *p* < 0.001), left ILF (F(1,80) = 51.929; *p* < 0.001) and right ILF (F(1,80) = 59.954; *p* < 0.001), left SLF (F(1,80) = 57.911; *p* < 0.001) and right SLF (F(1,80) = 61.; *p* < 0.001), left UNC (F(1,80) = 61.483; *p* < 0.001) and right UNC (F(1,80) = 57.729; *p* < 0.001) (Fig. 3).

Excluding the commissural tracts, Fmj and Fmn, hemispheric asymmetries in MD values were assessed by GLM Repeated Measure ANOVA using MD values (of each tract) and Hemisphere as repeated measures showed that exist a main effect of MD values (*F* = 14.643, df = 7, *p* < 0.001, ɛ = 0.476) and no effect for Hemisphere (*F* = 0.805, df = 1, *p* = 0.372, ɛ = 1); however, de double interaction FA values x Hemisphere was significant (*F* = 4.175, df = 7, *p* < 0.001, ɛ = 0.599).

Planned comparison showed that CGC (F(1,80) = 31.748; *p* < 0.001), CGH (F(1,80) = 15.253; *p* < 0.001), IFOF (F(1,80) = 97.012; *p* < 0.001), ILF (F(1,80) = 5.320; *p* < 0.023) have a right asymmetry (MD in the right hemisphere > MD in left hemisphere). No significant differences between hemispheres in the MD values, SLF and UNC were founded despite of their right asymmetry. On the contrary ATR (F(1,80) = 122.194; *p* < 0.001) and CST (F(1,80) = 7.333; *p* = 0.008) showed a left asymmetry.

## Discussion

### Reconstruction of the tracts of interest

The tridimensional reconstruction of ten tracts of white matter was achieved in a representative sample of Cuban population. The protocol included ROIs in a native space of each individual and the tractography method known as FACT. The trajectories obtained agree with neuro-anatomic descriptions derivate from post-morten and other tractographic studies [8, 21, 24, 25].

The ROIs that defined tracts’s trajectories were drawn on anatomical reference image from MNI steoreotaxic space according to the anatomical description reported for Mori et al. [8] and validated for Wakana et al. [7, 21]. Our data probed their reliability. These authors drawn the ROIs on each individual brain; however, we drawn the ROIs on MNI space and automatically were transformed to native space of each individual allowing the optimization of ROIs procedure and diminish the time needed for their analysis and the inter-subject variability. Also this analysis begins from the total tracking of the all brain, and posteriorly the tracts were select using the ROIs. This approach produces a nice balance of fibers density [26] along the resultant tract giving reliability to the results.

The tracking method used (FACT) has been previously validated in several studies but they mostly use this algorithm as a tool for study white matter tracts in pathological conditions (e.g. [27–29]). Only a few reports have been dedicated to standardize and reproduce a methodology using this method for study many tracts at the same time and in healthy subjects [7–9, 21]. In cases where this kind of study was made, the sample used was small (less than 30 subjects). In this work it was achieve to replicate the reconstruction of several white matter tracts in a wide sample of healthy subjects (*n* = 84).

### Characterization of reconstructed tracts

Previously, the parameters volume, FA and MD have been used to characterize white matter tracts [21, 30], being this paper a reference of these parameters in Cuban population.

The volume of studied tracts include a wide range of values (from five to 38 cm^3^) as has been describe anteriorly [31]. The values of FA can vary widely according to register parameters and methodology applied, however the values of this study (0.34˂FA˂0.58) agree with the range of values reported for this variable in white matter tracts reconstructed by Wakana et al. [21] (0.42˂FA˂0.60). The values of MD varied between 18 × 10^−5^ and 23 × 10^−5^, also were included in reported values [32].

All the reconstructed tracts show an inverse relationship pattern between FA and MD. This situation is not a trivial issue due to the MD value is similar between white matter of high anisotropy and gray matter of low anisotropy [33] moving into a narrow range of values. Only in case of cerebrospinal fluid take high values [34, 35] due to isotropic diffusion and free of barriers, producing high autovalues of diffusion tensor in all directions of water molecules [36, 37]; this phenomenon does not occur in gray matter where the water is restricted because of tissue properties. However, in white matter, the diffusion is anisotropic because of the packing of fibers and myelin presence [5], remaining MD low due to the small diffusion values in the perpendicular axis to the fiber directionality compensate that high diffusion in parallel axis to the fiber directionality. In this way, the mean of the autovalues of diffusion tensor remains low in white matter, because of that the MD can be used as a measure of presence of tissue. Otherwise, tracts constituted for fiber without an orientation pattern, myelin or packing structure will have low FA and high MD favoring the free diffusion. The unified using of FA and MD can be used as diagnostic tools in assess micro and meso-structural characteristic of brain tissue [38].

The tractography method FACT has some restrictions such as the acceptable angle between one voxel and the next one for a fiber trajectory, that can produce an underestimation in curved tracts (e.g. SLF and UNC) affecting the volume values. Also the volume estimation can be affected in fibers with bifurcation areas (e.g. IFOF) because the method can fail in estimate the way of the fibers. A presence of partial volume effects could affect the diffusion index due to mix of different kind of tissue in a voxel.

### Gender differences and hemispheric asymmetries

#### Volume

Several studies have demonstrated the existence of differences in hemispheric symmetry of the volume of gray matter structures and gender differences in the adult population [39–41]; however, there are limited studies that have addressed these issues to the white matter [21, 42].

Statistical analysis showed gender differences with larger volume in females than males for left and right ATR, right CGH, Fmj and Fmn, and right ILF. However, previously studies described a higher volume of white matter in males than females [43, 44]. In these studies, were compared the segmentations of white matter from T1 images and not the specific tract volume, therefore the differences in white matter volume between gender seems to be heterogeneous along each tracts of brain. Because of that, the study of specific tracts using DTI can be a more accurately approach to this matter. On the other hand, a global analysis of white matter volume shows bigger values in males [41], using correction for total intracranial volume, although was founded a paralleled slope for grey and white matter with cranial volume, whereas in women the increase in white matter as a function of cranial volume was at a lower rate. However, voxel-based morphometry studies revealed significant main effects of sex but no significant effects of brain size in white and gray matter analysis [45]. In spite of the fact that we did not find gender differences for SLF, there is a report about left asymmetry in males while in females this tract has a more bilateral distribution [46].

A decrement in commissural tracts (Fmj and Fmn) has been associated to a diminished interhemispheric connectivity with brain size, which can explain the less volume in males. This hypothesis is supported by studies of delay of information conduction and cellular cost of the process [47].

A left asymmetry was detected for ATR, CGC, CST, ILF and SLF; while CGH and IFOF showed a right one. The fact that a great number of tracts have bigger volume in left hemisphere agree with left dominance is expected in our right-handed sample. Our data showed a left asymmentry for tracts involve in motor control (CST) and language (SLF) [48–51]. This left asymmetry for CST has been previously reported by Rademacher et al. [52] and Thiebaut de Schotten et al. [46] and confirmed by post-mortem studies [31], but White et al. [53] suggest that there is not such asymmetry. Also, left asymmetry in volume of SLF [46, 54], CGC and ILF [21] has been reported previously. On the contrary, there is evidence of no significant differences for ATR between hemispheres [21]. Moreover, have been described a right lateralization for CGH [21] and IFOF [21, 46] as same in our results. These facts suggest that right hemisphere seems to be specialized in more general functions that require integration of information, such as visual-spatial processing [55]. Specifically, CGH is involve in memory associative learning and episodic processing [56], while IFOF connects functional areas of visuo-spatial processing [57]. The UNC did not show differences between hemispheres, however in literature there are conflicting reports. Highley et al. [58] found that this fasciculus had bigger volume in right hemisphere, while Wakanana et al. [21] described left asymmetry. This inconsistency in the results may be due to different methodologies employed or small samples (*N* < 30).

In our analysis we included age as continuous predictor but we did not find any effect of that factor, probably because of the sample age is very homogenous and included mostly young adults. Nevertheless, several reports evince a global white matter volume increase from childhood to adultness [59, 60], and a further declination after maturation [61]. Generally, aging is associated with a reduction in white matter volume [62, 63] that seems to be more pervasive at times than even the gray matter decline [64], and generally involve a reduction in the integrity of white matter tracts [65, 66]. Also, it has been reported that males had more prominent age-related gray matter decreases and white matter volume and corpus callosal area increases compared with females what suggest that there are age-related sex differences in brain maturational processes [67].

#### Fractional anisotropy and mean diffusivity

In previous studies have been described differences by gender in FA in specific tracts [21, 30, 32], meanwhile our data exhibited greater values of FA for all tracts in females. Sexual dimorphism has been demonstrated in microstructural white matter organization in precentral, cingulate, and anterior temporal areas, but reporting lower values for females [68]. In specific tracts in females such as CST, which is involved in motor function, have been reported highest values, while males may undergo relatively more microstructural change in projection and association fibers [69]. In another hand, Schmithorst et al. [70] had reported greater FA values in females for Fmj and Fmn, however when the entire corpus callosum has been studied the males happen to have the greatest values.

The FA showed significant left asymmetries for CGC, CGH, IFOF and ILF. Greater values of FA on lefts hemisphere have been describe previously for CGC [21], IFOF [21, 30, 71], ILF [21, 71], while CGH reported significant right asymmetry [21]. This lateralization was associated with higher microstructural integrity on the left side of limbic tracts (CGC and CGH) [72]. The ATR and CST had right asymmetries, which means in the ipsilateral hemisphere to handedness. That is an unexpected find because does not agree with postulation that right handed subject must have greater values of FA in contralateral hemisphere [49, 50], where have been observed better packing and arrangement of fibers that are involve in voluntary control of movement in contralateral hemisphere [40]. However the values are include in reported data for this index [30].

The data showed bigger values of MD for males than females in all the tracts, an inverse behavior than FA values. This higher MD values for females have been reported since adolescence for ILF and Fmj [69] and for CST in males [70]. However, in a study performed by Eluvathingal et al. [71] was not found gender effect over MD for any tracts except ILF, where girls had it lower values than males. The MD showed significant right asymmetries for CGC, CGH, IFOF and ILF, that means MD values have an inverse pattern than FA according to hemispheric asymmetries, besides gender asymmetry. The CGC and CGH have been previously reported with right-greater-than-left MD values, which remain for CGC even after normalization procedure [72]. Also data from IFOF and ILF have been reported with this asymmetry [71]. Besides the variety of reports some studies analyzing MD have revealed that it is a sensitive measure when were compared controls and pathological subjects [73]. No difference between hemispheres or gender for SLF and UNC were found, either FA or MD measures. This does not agree with previous results were a right-higher-than-left anisotropic asymmetry was found [74] for these tracts, however Büchel et al. [40] reported a left asymmetry for SLF. Also Kitamura et al. [75] had detected gender difference in the FA values for the right UNC.

As well as in statistical analysis of volume, neither FA or MD showed any effect of age probably due to the age of the sample is around young adultness. However, these index have been used to explain the changes in white matter integrity that occurs across life spam. Previous reports of DTI studies have shown an increased FA [76] throughout brain white matter during childhood, adolescence, and young adulthood [71, 77–79] and later elderly adults have displayed a significant decline in several white matter tracts [80]. On the other hand, Inano et al. [81] suggest there are no sex differences in the aging process of the white matter in a sample with an age range from 24.9 to 84.8 years.

## Limitations

The resolution is limited due to the voxel size employed in diffusion imaging series. Also it is possible the modification of diffusion indexes by noise, partial volume effect and crossing fibers regions, which can miss estimate the parameters.

The tract volume was not normalized according to intracranial volume. However, is postulated that differences in hemispheric symmetry and gender are not modified for normalization procedure [21, 82].

## Conclusions

Our work shows that is possible reconstruct white matter tracts using a unique ROIs scheme defined on a standard space, that can be transformed automatically to individual anatomy, minimizing the effect of investigator’s manipulation. Also, allows the creation of a database of volume and diffusion parameters in Cuban population that can be used as normative sample in others studies. The volume values showed that a most part of tracts have bigger volume in left hemisphere. The data showed bigger values of MD for males than females in all the tracts, an inverse behavior than FA values. These results indicate differing developmental trajectories in white matter for males and females and the importance of taking gender into account in developmental DTI studies and in underlie gender-related cognitive differences. This study will provide the opportunity to analyze gender-specific nature of brain diseases supported by a control sample that allows the comparison between normal and pathological status.

## Additional file

Additional file 1: Supplementary results. (DOCX 2185 kb)

## Abbreviations

ATR: Anterior thalamic radiation
CGC: Cingulate gyrus associated cingulum
CGH: Hippocampal gyrus associated cingulum
CST: Corticospinal tract
DTI: Diffusion tensor imaging
EPI: Echo planar Imaging
ET: Echo time
FA: Fractional anisotropy
FACT: Fast assignment by continuous tracking
Fmj: Forceps major
Fmn: Forceps minor
FOV: Field of view
GLM: General linear model
IFOF: Inferior fronto-occipital fasciculus
ILF: Inferior longitudinal fasciculus
IQ: Intelligence quotient
MD: Mean diffusivity
MNI: Montreal Neurological Institute
rmANOVA: Repeated measure analysis of variance
ROI: Region of interest
RT: Repetition time
SLF: Superior longitudinal fasciculus
UNC: Uncinate fasciculus
