# Supplementary material for: Characterization of ten white matter tracts in a representative sample of Cuban population
Source: BMC Med Imaging. 2016 Oct 26;16:59. doi: 10.1186/s12880-016-0163-7 (PMC5082362; doi:10.1186/s12880-016-0163-7)
Supplement: Additional file 1: — Supplementary results. (DOCX 2185 kb) [file 12880_2016_163_MOESM1_ESM.docx]

**SUPPLEMENTARY MATERIAL**

*Results*

We were able to reconstruct ten white matter tracts using the deterministic method FACT and ROIs obtained for each subject by the transformation proceeding of reconstruction of the trajectory proposed for ten tracts of interest in each of the 84 subjects enrolled in the study. The three-dimentional reconstruction of these tracts were performed separately four functional categories: brainstem fibers and projection (Figure 1), association fibers (Figure 2 of Supplementary Results), tracts of the limbic system (Figure 3) and commissural fibers (Figure 4).

In the first category, brainstem fibers and projection are included the cortico-spinal tract and anterior thalamic radiation, whose reconstructions are illustrated in Figure 1 on a subject of the sample under study. CST crosses the brainstem and reaches pre and postcentral gyrus. ATR fibers converge at the inner capsule, which is located between putamen and thalamus and caudate nuclei regions (Figure 1).


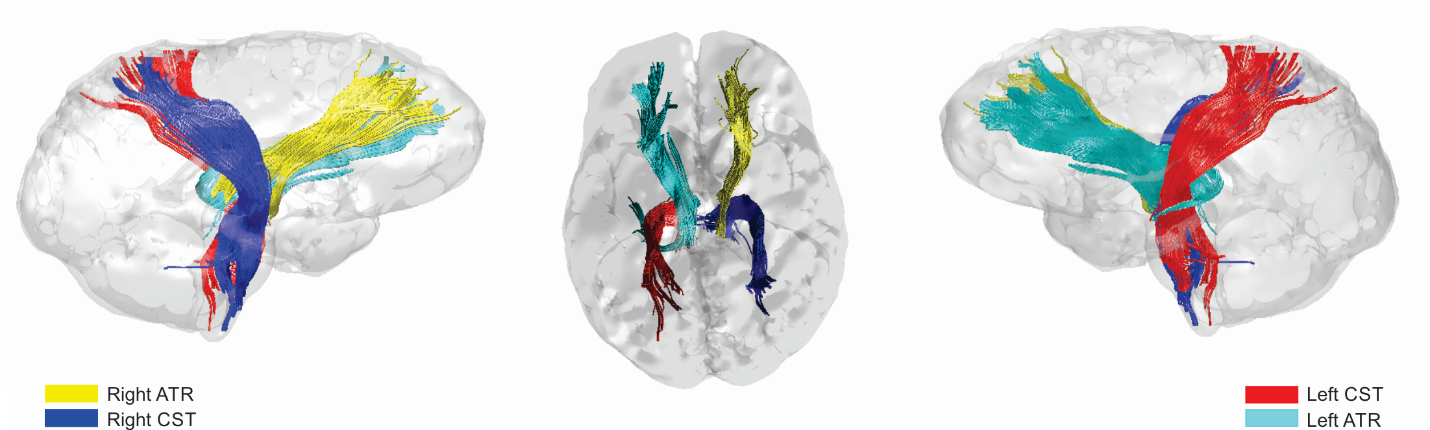
**Fig. 1** Three-dimentional reconstruction of brainstem and projections fibers: cortico-spinal tract (CST) and anterior thalamic radiation (ATR) in right lateral view (left panel), superior view (central panel) and left lateral view (right panel).

Also, it was traced the path of four association tracts well documented in the literature. They were ILF, IFOF, the SLF and UNC. Reconstruction obtained from these tracts is illustrated in Figure 2, on a subject of the sample under study. Here the parallel path of the ILF and IFOF is evident, which share projections in the occipital lobe and posterior temporal lobe. The SLF projects towards most lateral temporal lobe regions with a characteristic C-shaped. The SLF is the only association tract projecting its fibers into the thalamus and throughout the ventricle. The UNC connects the temporal lobe with the frontal lobe in each hemisphere. It can be appreciated that UNC share projections in the frontal lobe with the IFOF.


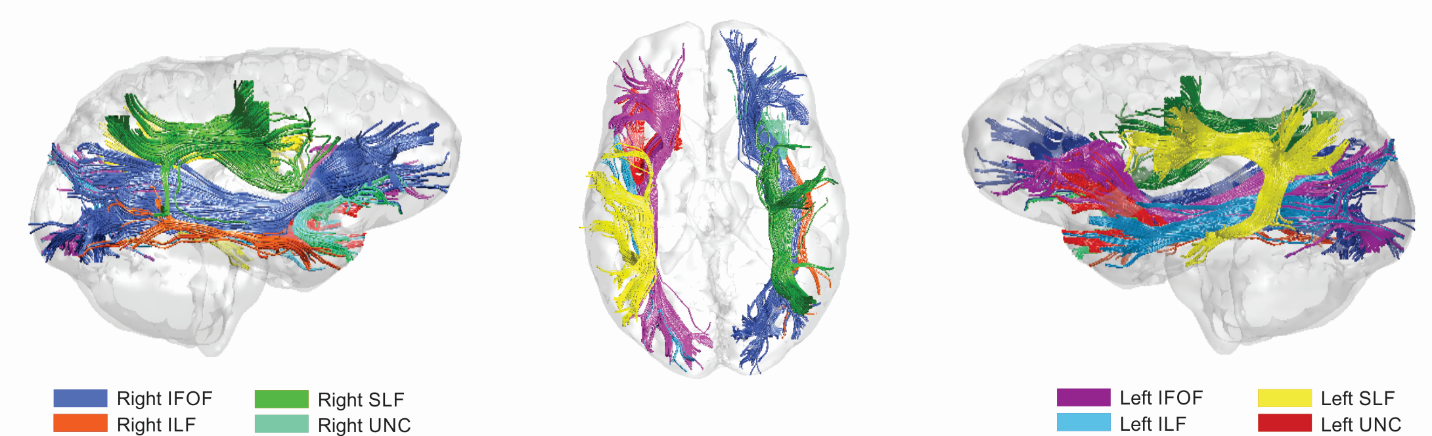
**Fig. 2** Three-dimentional reconstruction of association fibers: inferior fronto-occipital fasciculus (IFOF), inferior longitudinal fasciculus (ILF), superior longitudinal fasciculus (SLF) and uncinate fasciculus (UNC) right lateral view (left panel), superior view (central panel) and left lateral view (right panel).

Belonging to the limbic system, the cingulate bundle was rebuilt into two branches, one associated with the cingulate gyrus and the other to the hippocampus (Figure 3). The CGC collected fibers from cingulate gyrus that extend to the temporal lobe, and the CGH runs the ventral surface of the hippocampus.


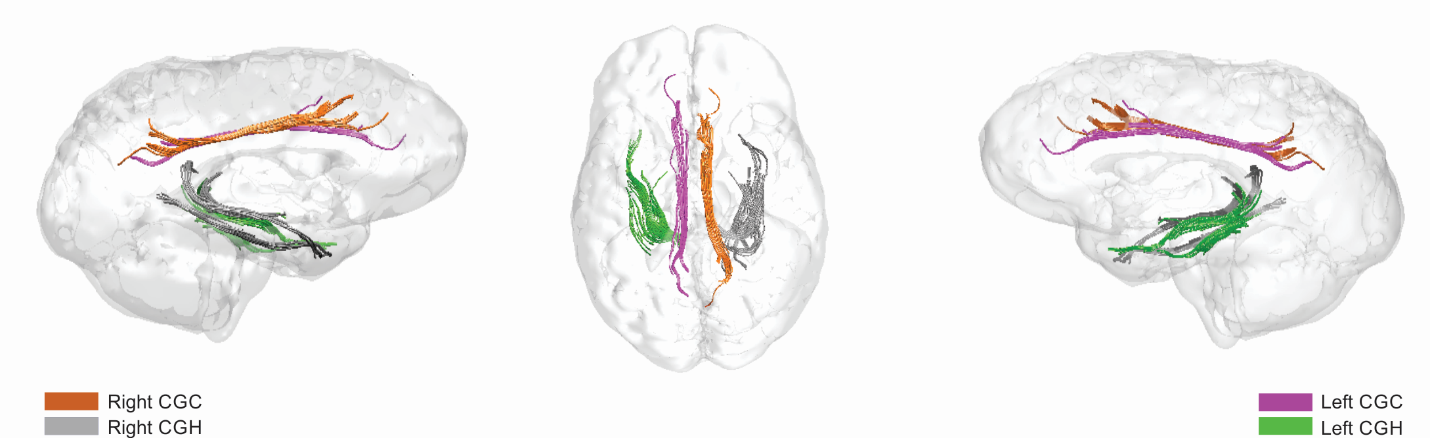
**Fig. 3** Tridimentional reconstruction of limbic system tracts: cingulate gyrus associated cingulum (CGC) and hippocampal gyrus associated cingulum (CGH) in right lateral view (left panel), superior view (central panel) and left lateral view (right panel).

In the category of commissural fibers were reconstructed the forceps minor and major shown in Figure 4. The forceps minor is formed by the projections that connect the frontal lobes through the genu of the corpus callosum and the forceps major by the communicating fibers of the occipital lobes crossing the splenium of the corpus callosum.


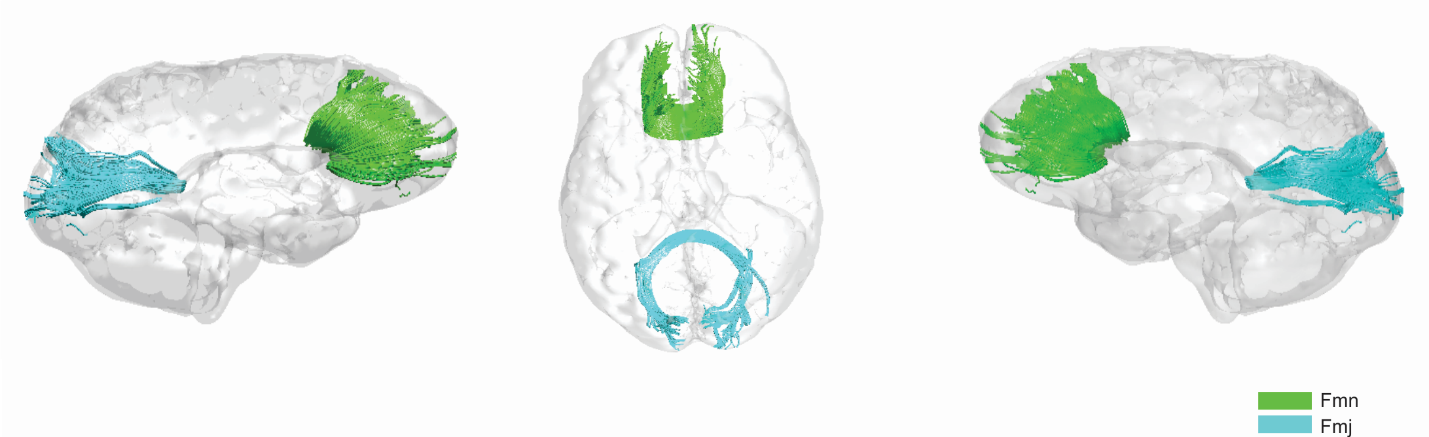
**Fig. 4** Three-dimentional reconstruction of commissural fibers: forceps major (Fmj) and forceps minor (Fmn) in right lateral view (left panel), superior view (central panel) and left lateral view (right panel).
